# Engineering digital biomarkers of interstitial glucose from noninvasive smartwatches

**DOI:** 10.1038/s41746-021-00465-w

**Published:** 2021-06-02

**Authors:** Brinnae Bent, Peter J. Cho, Maria Henriquez, April Wittmann, Connie Thacker, Mark Feinglos, Matthew J. Crowley, Jessilyn P. Dunn

**Affiliations:** 1 Department of Biomedical Engineering, Duke University, Durham, NC USA; 2 Department of Statistical Science, Duke University, Durham, NC USA; 3 Division of Endocrinology, Department of Medicine, Duke University Medical Center, Durham, NC USA; 4 Department of Biostatistics and Bioinformatics, Duke University Medical Center, Durham, NC USA

**Keywords:** Biomedical engineering, Biomarkers, Pre-diabetes

## Abstract

Prediabetes affects one in three people and has a 10% annual conversion rate to type 2 diabetes without lifestyle or medical interventions. Management of glycemic health is essential to prevent progression to type 2 diabetes. However, there is currently no commercially-available and noninvasive method for monitoring glycemic health to aid in self-management of prediabetes. There is a critical need for innovative, practical strategies to improve monitoring and management of glycemic health. In this study, using a dataset of 25,000 simultaneous interstitial glucose and noninvasive wearable smartwatch measurements, we demonstrated the feasibility of using noninvasive and widely accessible methods, including smartwatches and food logs recorded over 10 days, to continuously detect personalized glucose deviations and to predict the exact interstitial glucose value in real time with up to 84% and 87% accuracy, respectively. We also establish methods for designing variables using data-driven and domain-driven methods from noninvasive wearables toward interstitial glucose prediction.

## Introduction

There is currently no commercially-available noninvasive method for monitoring glycemic status; specifically, noninvasive glucose monitoring to inform glycemic self-management, particularly for prediabetics, is lacking. Prediabetes affects over one-third of people in the United States^1^. While prediabetes is highly prevalent and has serious consequences, it is also seriously underdiagnosed—only ten percent of people with prediabetes are aware that they have the disease^2^. For those who have been diagnosed, prediabetes is often poorly managed^3–5^, leading to 70% of individuals with prediabetes to eventually develop type 2 diabetes (T2D) and to a 10% annual conversion rate from prediabetes to T2D^6^. Prediabetes is reversible with lifestyle modifications: the Diabetes Prevention Program reduced diabetes incidence by 58% through interventions aimed at weight loss, dietary change, and physical activity in patients with prediabetes^7^. Recently, monitoring of blood glucose levels has been added to several prediabetes treatment plans^8,9^ and tracking of blood glucose is even being used by those with normal glucose levels in order to better understand and track glycemic and metabolic health^10,11^. Long-term lifestyle changes are more likely when patients self-monitor their blood glucose^12^, and this practice can be upheld by easily accessible methods for glycemic health monitoring that aid in self-management of prediabetes.

Glucose control is often a main objective of diabetes management, which includes regulating glucose variability and avoiding glucose deviations. Definitions of glucose deviations, including ‘hyperglycemia’ (glucose that is too high) and ‘hypoglycemia’ (glucose that is too low), have been widely cited in literature pertaining to type 1 diabetes (T1D) and T2D^5,13–24^. Hyperglycemia is traditionally defined as having non-fasting glucose above 180 mg/dL and hypoglycemia is traditionally defined as having non-fasting glucose below 70 mg/dL^25^. These definitions were developed for diabetes management^19^. However, these may not be adequate to explain glucose deviations in individuals with prediabetes and normal hemoglobin A1c (HbA1c) due to lower fasting glucose levels and lower glucose variability than in those with diabetes. The importance of personalization of glycemic health has been demonstrated previously using ‘glucotypes’ to describe intraindividual differences in glycemic responses^26^. Management of glucose fluctuations begins with an understanding how specific behaviors influence a person’s own blood glucose levels. Current methods for monitoring blood glucose^27^, including blood glucose meters and continuous glucose monitors, are not frequently utilized by non-insulin-dependent patients due to their inconvenience, invasiveness^28^, and high cost. There is currently no commercially-available noninvasive tool to estimate interstitial glucose in real-time.

Non-invasive wrist-worn biometric monitoring technologies^29^, often referred to as ‘wearables’^30^, are becoming nearly ubiquitous in the United States, with 117 million currently in use and an expected 100% growth in the next three years^31^. Because of this widespread use, wearables can enable digital biomarker discovery which will facilitate detection and monitoring of chronic diseases^30^. Digital biomarkers are digitally collected data (e.g., heart rate measurements from a wearable) that may be used as indicators of health outcomes (e.g., prediabetes)^32^. Digital biomarker algorithms support the aggregation of high-resolution, intra-individual data into summary metrics that are interpretable and actionable^33^.

There are many competing factors that affect blood glucose levels, including diet^34^, activity and exercise^35^, stress^36^, circadian rhythm^37^, and biological sex^38^. There are also factors that are related to glycemic health and glucose fluctuations, although they may not affect blood glucose directly, including heart rate^39^, body temperature^40^, and autonomic functions^41^ including the sudomotor response^42^. The factors affecting and relating to blood glucose are extremely personalized: individualized factors can have significant impacts on the blood glucose dynamic^25^, glycemic responses to foods are highly individual, and the foods that raise a person’s blood glucose can vary dramatically^43^.

**Fig. 1:**
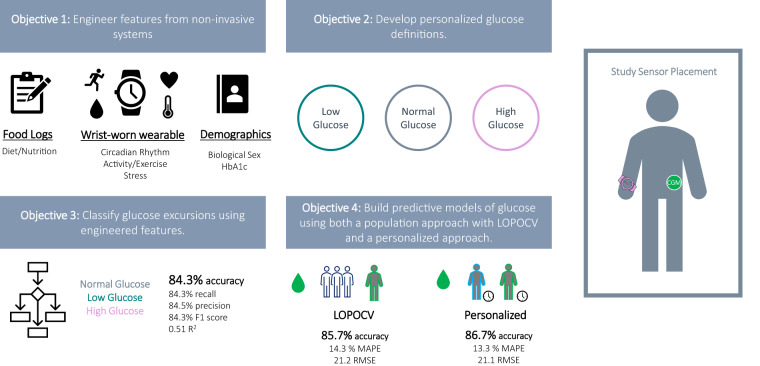
Graphical abstract of study. This study has four objectives: (1) Engineer features from non-invasive systems using a combined data-driven and domain-driven feature engineering approach. (2) Develop personalized glucose excursions definitions. (3) Classify glucose excursions using engineered features. (4) Build predictive models of glucose using both a population approach with leave-one-person-out cross validation and a personalized approach. Sensor placement for the study (Empatica E4 on the wrist and a Dexcom G6 continuous glucose monitor on the abdomen) is also shown.

The primary objective of this study is to determine whether we can build models from noninvasive wearables data combined with food logs to classify interstitial glucose levels and to predict interstitial glucose (Fig. 1). This would enable a frictionless and noninvasive method to monitor interstitial glucose in real time, allowing patients to engage with their glycemic health and actively monitor their progress while employing lifestyle modifications.

## Results

### Developing personalized definitions of interstitial glucose excursions for detecting intraindividual excursions

**Fig. 2:**
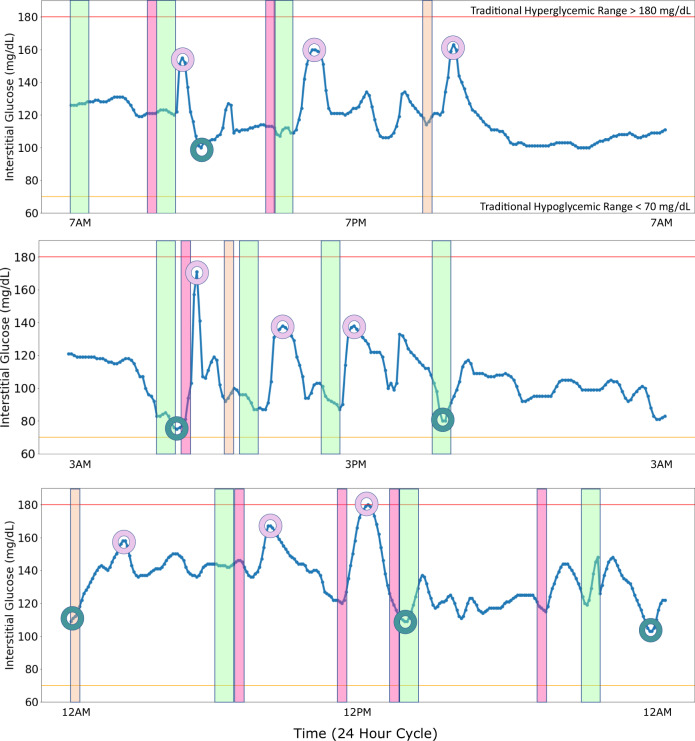
Case studies of CGM data over 24 h for 3 participants highlighting eating habits and subsequent glucose excursions. Green bands indicate a meal or snack, pink bands indicate consumption of a sugary beverage or soda, and orange bands indicate a sugary snack or dessert. Shown in red is the traditional hyperglycemic range (>180 mg/dL) and shown in yellow is the traditional hypoglycemic range (<70 mg/dL). Note that the participants do not exceed the traditional definitions of glucose excursions. A system that would alert prediabetes patients based on traditional definitions of glucose excursions would be inadequate in these cases. The personalized classifications we propose in this paper would better inform prediabetes patients so they can begin to self-manage their diet, exercise, and stress levels based upon this information. As shown, the personalized classes PersHigh (shown in plum) and PersLow (shown in teal) would provide more personalized information about fluctuating glucose. For example, sugary drinks and snacks result in higher glucose fluctuations. By reducing the amount and frequency of sugary drinks and snacks, participants would be less likely to experience a glucose excursion.

**Fig. 3:**
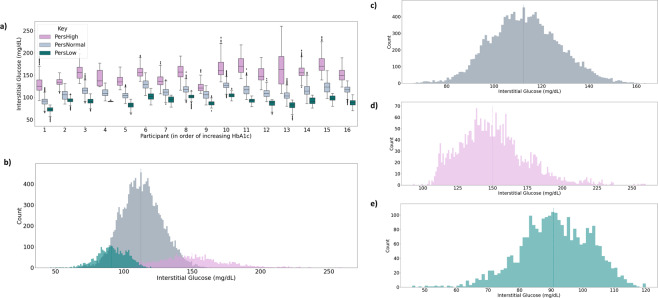
Personalized glucose excursions. Glucose excursions are classified on a personalized, rolling basis examining the previous 24 h of each participant’s historical data. **a** Boxplots of PersNorm, PersHigh, and PersLow for each participant in the dataset. **b** Histogram of all distributions over all participants. **c** PersNorm distribution. **d** PersHigh distribution. **e** PerLow distribution. Bin width for each histogram shown is 1 mg/dL.

Study participants were 35–65 years of age with elevated blood glucose in the normal range (HbA1c 5.2–5.6) or prediabetes (HbA1c 5.7–6.4) (Supplementary Table 2). Existing clinical definitions of blood glucose excursions, including ‘hyperglycemia’ (glucose that is too high) and ‘hypoglycemia’ (glucose that is too low), are defined at the population level and are largely used to describe glucose excursions in T1D and T2D patients^5,13–19^; however, these classifications were established for diabetes management^19^ and may not be suitable to explain significant glucose excursions in normoglycemic or prediabetic individuals due to their lower overall fasting glucose levels and lower glucose variability as compared with diabetic individuals. Examples demonstrating this scenario are shown in Fig. 2, where three separate participants had clear glycemic responses to specific behaviors, including sugary food or drink intake (spike in blood sugar) or not eating (drop in blood sugar). These excursions were not sufficiently high or low as to be clinically categorized as hyper- or hypoglycemia and would therefore be considered normal as per the traditional definitions, even though they are high or low compared to the individual’s own baseline. Personalized glucose excursion classes (Fig. 2 circles) provide more tailored information about glucose fluctuations and enable self-management and tracking of diet, exercise, and stress-related behaviors that affect blood glucose. Furthermore, the glycemic baseline is also dynamic over time^44–46^, so single-valued population-level thresholds to define glucose excursions are inadequate for understanding an individual’s deviations over time from their typical state^25,26,43^. Thus, we developed three personalized and dynamic designations to categorize each interstitial glucose measurement to indicate the presence and absence of personalized glucose excursions. We denote these categories as PersHigh, PersLow, and PersNorm, which correspond to an interstitial glucose measurement that is greater than, less than, or within one standard deviation of the 24-h personalized mean, respectively (Fig. 3a). These personalized and time-varying calculations account for circadian, intra-, and inter-day variability. Each of the three categories are approximately normally distributed (PersNorm Kolmogorov–Smirnov Normality Test (KS) Statistic=0.03 (Fig. 3c); PersHigh KS Statistic=0.05; PersLow KS Statistic=0.04) (Table 1). The PersHigh distribution is skewed moderately right and is leptokurtic, with more data located at the tails of the distribution rather than around the mean (Fig. 3d, Table 1). PersLow is skewed slightly left and is mesokurtic, with a distribution moderate in breadth and approximately normally distributed (Fig. 3e, Table 1). The wider distribution for PersHigh compared to PersLow likely reflects the fact that there is a wider range of possible hyperglycemic values than hypoglycemic values in our population. Interestingly, there is an overlap in all three distributions between interstitial glucose values of 66–164 mg/dL (Fig. 2b), supporting the idea that what may be considered a normal measurement for one person may actually be a low or high measurement for another person, which also points to the inadequacy of population-level thresholds.

**Table 1:** Summary metrics of distributions of each personalized glucose definition.

Glucose class	Mean (mg/dL)	Standard deviation (mg/dL)	Range (mg/dL)	Skewness	Kurtosis
PersNorm	112.4	14.5	66.0 - 164.0	0.12	−0.10
PersHigh	149.9	24.4	93.0 - 260.0	0.75	0.93
PersLow	90.8	11.1	46.0 - 120.0	−0.32	0.17

The categories PersHigh, PersLow, and PersNorm are personalized glucose definitions that correspond to an interstitial glucose measurement that is greater than, less than, or within one standard deviation of the 24-h personalized mean, respectively.

### Glucose excursion classification

For many patients with conditions like prediabetes, simply understanding which behaviors trigger high or low glucose excursions would greatly improve disease management. A number of measurable factors influence blood glucose levels, including diet^34,47–57^, physical activity and exercise^35,58–62^, stress^36,63–69^, circadian rhythm^37,55,70–73^, and biological sex^38,74–80^. Additionally, physiological parameters like vital signs are associated with glycemic health and glucose fluctuations, including heart rate^39,81,82^, core body temperature^40,83^, and autonomic functions^41,84–86^ like the sudomotor response^42^. These relationships suggest that it may be possible to estimate glucose values from novel modes of measurement. To determine whether this is possible, we developed classification models using data from these alternative modes of measurement with the goal of detecting when interstitial glucose was outside of the personal norm.

We engineered 69 variables based on the previously described literature^34–42,47–86^ as inputs into our prediction models using a combination of data-driven and domain-driven feature engineering (Supplementary Table 1). These variables were built using data collected from a noninvasive wearable, a food log, and electronic reports of demographics. Measurements included metrics of stress, circadian rhythm, diet, activity and exercise, heart rate, skin temperature, and biological sex.

**Fig. 4:**
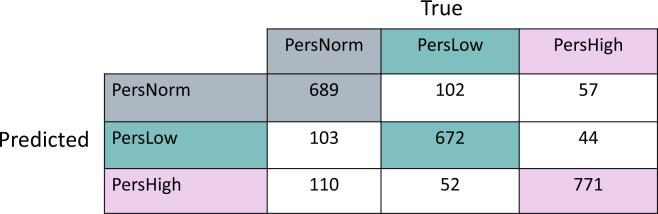
Confusion matrix: multiclass model. Confusion Matrix for multiclass decision tree model validated with a 70/30 train/test split. In this model, the class-accuracy for PersHigh glucose is 82.6%, the class accuracy for PersNorm is 81.3%, and the class accuracy of PersLow is 82.1%.

We defined the ground truth for intraindividual excursions, classified as PersHigh, PersLow, or PersNorm, based on a personalized, rolling basis from CGM measurements. We developed a multi-class model to classify between PersLow, PersHigh, and PersNorm interstitial glucose using a class-balanced dataset (*N* = 8666). The decision tree classifier with repeated stratified k-fold cross validation achieved an accuracy of 84.3 ± 0.013% (recall = 84.3 ± 0.013%; precision=84.5 ± 0.013%; weighted F1 Score=84.3 ± 0.013%; *R*^2^ = 0.505 ± 0.050) (Table 2). We repeated this modeling task using a 70/30 train/test (TT) split. The decision tree classifier using the 70/30 TT split achieved 82.0% accuracy (recall = 82.0%; precision=82.3%; F1 Score=82.1%; *R*^2^ = 0.46) (Table 2). The confusion matrix for the decision tree classifier using the 70/30 TT split is shown in Fig. 4. The per-class accuracies for each of the three interstitial glucose classes were similar: the class accuracy for PersHigh glucose was 82.6%, the class accuracy of PersNorm was 81.3%, and the class accuracy for PersLow was 82.1%. The decision tree models both outperformed logistic regression (accuracy = 52.0%; recall=52.0%; precision=52.3%; F1 Score=52.0%; *R*^2^ = 0 (Method of calculation for R^2^ enabled negative values, which were thresholded at zero), indicating that more complex relationships in the data need to be captured to perform highly accurate classification (Table 2).

**Table 2:** Model evaluation metrics for multi-class models.

Model	Balanced accuracy	Recall	Precision	F1 Score	*R*^2^
Multiclass Decision Tree Repeated Stratified K-Fold CV	84.3 ± 0.013%	84.3 ± 0.013%	84.5 ± 0.013%	84.3 ± 0.013%	0.505 ± 0.050
Multiclass Decision Tree 70/30 Train/Test Split	82.0%	82.0%	82.3%	82.1%	0.455
Multiclass Logistic Regression 70/30 Train/Test Split	52.0%	52.0%	52.3%	52.0%	0^a^

^a^Method of calculation for R^2^ enabled negative values, so the threshold was set to 0.

### Glucose prediction

Prediction of precise interstitial glucose values as opposed to whether or not a person is experiencing a high or low excursion would give additional information for glucose self-monitoring and tracking. Thus, we extended our models to determine whether noninvasive wearables could serve as a proxy for a continuous glucose monitor. In order to predict glucose at 5-min intervals, we developed both a gradient-boosted population model validated with leave-one-person-out cross validation (LOPOCV) and a gradient-boosted personalized model, trained and tested on each individual’s previously measured data. Our population model validated with LOPOCV had an average root mean squared error (RMSE) of 21.22 ± 4.14 mg/dL and an average mean average percent error (MAPE) of 14.33 ± 3.25%. Average accuracy for the population model over all participants was 85.67%. Using the initial half of the participant’s data for training, the personalized model trained and tested on each participant’s data had an average RMSE of 21.10 ± 4.50 mg/dL and an average MAPE of 13.26 ± 3.94%. Average accuracy for the personalized model across all participants was 86.74%. Both the personalized model and the traditional population model outperformed the naïve models (mean and median). That the population and personalized models performed comparably may indicate that there are common factors that are mostly sufficient for modeling.

### Exploring feature importance for glucose prediction

**Fig. 5:**
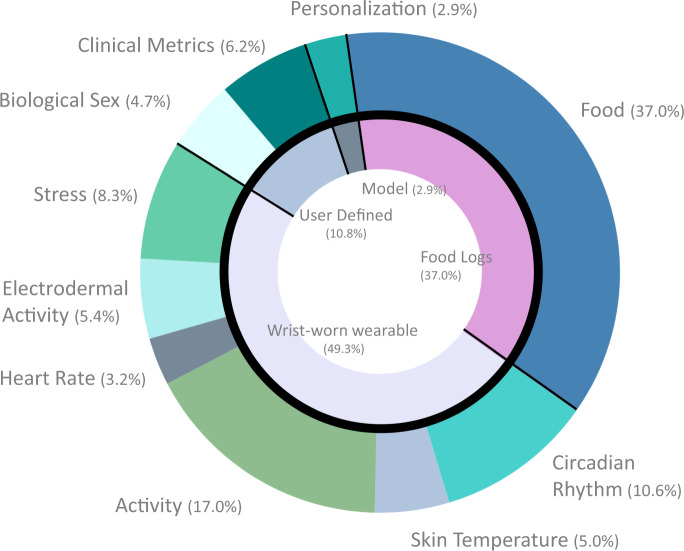
Importance of 69 domain-driven and data-driven features in glucose predictions. Importance was determined from a random forest feature selection model using impurity-based features. The outer circle shows relative percent of importance for the categories food, circadian rhythm, activity, stress, gender, clinical metrics, personalization, electrodermal activity, heart rate, and skin temperature. The inner circle shows relative importance by source of features, including food logs, the wrist-worn wearable, user-defined features, and features defined for/by the model.

In order to determine the contribution of each of the 69 variables to the interstitial glucose prediction, we calculated impurity-based importance for each variable in the LOPOCV random forest regression models with importances averaged across each fold. The feature importances allow us to examine the extent that each measurement type contributed to the success of the model and therefore serve as a mechanism to generate hypotheses of potential physiologic relationships that can be tested directly through experimentation. Features were aggregated together into the following categories: ‘food’, ‘circadian rhythm’, ‘stress’, ‘activity’, ‘temperature’, ‘heart rate’, ‘electrodermal activity’, ‘biological sex’, ‘HbA1c’, and ‘personalization’. They were further categorized by the source of the data: ‘food log’, ‘wearable’, ‘user input’, and ‘model’. Based on the literature we anticipated that the most important features would be related to food, activity, circadian rhythm, and stress^34–37,47–73^ and found that indeed food had the highest importance, with an average of 37.0% (percent of total importance, where total importance sums to 100%), followed closely by activity (17.0%), circadian rhythm (10.6%), and stress (8.2%). Of the feature importances, 49.3% were derived from a wearable, 37.0% were sourced from the food log, 10.8% were user input (including biological sex and HbA1c), and 2.9% were personalization features of the model. This supports further development of multi-modal models using features from both a noninvasive wearable and a food diary to predict glucose. In terms of how the features were engineered, 66.8% of feature importances were domain-driven, 19.5% were data-driven, and 13.7% were neither (demographics data) (Fig. 5).

The fifteen most important features in the model (mean impurity-based feature importance >0.02), ordered from most to least important, included measures of circadian rhythm, diet, demographics, exercise/activity, and stress (Table 3, Supplementary Fig. 1). The variance among the LOPOCV models is illustrated in Supplementary Fig. 1 and highlights the variability in the most important features.

**Table 3:** Exploring feature importance for glucose prediction: the 15 most important features in the random forest model.

Feature	Feature category	Impurity-based feature importance (mean ± standard deviation)
Minutes from midnight	Circadian rhythm	0.069 ± 0.004
Sugar intake (previous 24 h)	Food	0.067 ± 0.038
Hemoglobin A1c (HbA1c)	Clinical/Demographics	0.062 ± 0.018
Carbohydrates (previous 2 h)	Food	0.047 ± 0.016
Biological sex	Demographics	0.047 ± 0.033
Activity (previous 24 h)	Activity/Exercise	0.042 ± 0.005
Sugar intake (previous 2 h)	Food	0.038 ± 0.022
Sugar intake (previous 8 h)	Food	0.031 ± 0.004
Accelerometry (mean previous 2 h)	Activity/Exercise	0.030 ± 0.002
Wake time	Circadian rhythm	0.029 ± 0.005
ID	Personalization	0.029 ± 0.016
Accelerometry (max previous 2 h)	Activity/Exercise	0.026 ± 0.002
Protein (previous 24 h)	Food	0.025 ± 0.002
Peak electrodermal activity (mean previous 2 h)	Stress	0.024 ± 0.009
Carbohydrates (previous 24 h)	Food	0.024 ± 0.003

Overall, novel findings from this work included the development of personalized definitions of interstitial glucose excursions, demonstrating robust classification of these personalized interstitial glucose excursions from non-invasive wearables and food logs data and creating a noninvasive continuous glucose monitor “proxy” with high accuracy. We developed novel feature engineering methods that achieve high accuracy in interstitial glucose classification and regression models. These features highlight the existence of important relationships between physiologic measurements from non-invasive wearables and interstitial glucose fluctuations which serve as a basis for future experimental studies.

## Discussion

The primary objective of this study was to determine whether we can use noninvasive wearables with food diaries to detect personalized interstitial glucose excursions and predict interstitial glucose values. We integrated data-driven and domain-driven feature engineering methods to engineer 69 features, and we developed a personalized, rolling baseline to determine glucose excursions. We used our engineered features in machine learning models to classify glucose excursions. Finally, we used our engineered features to develop and compare population-level and personalized models for glucose prediction.

Typically, patients with diabetes or other glucose metabolism conditions monitor their glucose by measuring their blood sugar level periodically throughout the day with a blood glucose meters or continuous glucose monitors, both of which involve an invasive needle and are not typically recommended for individuals with prediabetes. Innovative, practical strategies to improve monitoring and management of glycemic health are desperately needed. One way we can improve management of glycemic health is by using noninvasive wearables and food logging to determine when a participant is experiencing interstitial glucose excursions (high glucose or low glucose).

There is a lack of prediabetes-specific thresholds for glucose deviations, which makes prediabetes monitoring and disease management challenging. Furthermore, the spectrum of prediabetes is highly personalized^26,43^ and personalized thresholds for glucose excursions do not currently exist, preventing personalized monitoring. To address this gap, we developed personalized definitions of interstitial glucose excursions. Defining glucose excursions on a personalized, rolling basis enables the use of a participant’s historical data to better understand deviations from a personalized baseline over a defined window of time. This also allows accounting for intraday variability in addition to interday variability.

Here, we developed a multi-class model to detect PersHigh, PersLow, and PersNorm interstitial glucose events that achieved 84.3% balanced accuracy. Currently, these classification models could be employed in practice to alert patients with prediabetes when they may be experiencing a glucose excursion to check their blood glucose. Overall, optimizing the timing of blood glucose checks will better inform people about the effect of their behaviors and lifestyle habits on their blood glucose. For example, a patient’s own historical data can show them how reducing the amount and frequency of sugary drinks and snacks would reduce the likelihood of a glucose excursion. In the future, further validation of these models may enable their use alone without additional tools to self-monitor and track blood glucose.

Displaying exact glucose values to users has been shown to improve logical reasoning and influence their eating and activity behaviors^87^, which could be directed toward improved glucose control. Having specific, numeric, goals have been demonstrated to increase and maintain motivation^88^. Here, we demonstrate the feasibility of predicting glucose from non-invasive data at 5-min intervals (the same sampling rate as a continuous glucose monitor) which could further enable patients to understand how their lifestyle habits are influencing their blood glucose levels and help them manage their disease. Interestingly, while continuous glucose monitors are widely used to inform insulin delivery, their accuracy can vary substantially from a blood glucose meter. For example, the Dexcom G6 is considered sufficiently accurate if its measurements fall within ±20 mg/dL of a simultaneous blood glucose meter measurement for meter values <100 mg/dL and ±20% for meter values >100 mg/dL^89^. As such, the accuracy of our newly proposed noninvasive glucose prediction models is within the accuracy realm of currently used technologies (RMSE = 21.22 ± 4.14 mg/dL).

The random forest-based glucose prediction models showed that, of the top features, 49.3% were derived from a wearable, 37.0% were sourced from the food log, 10.8% were from user inputs (including biological sex and HbA1c), and 2.9% were resulting from the model itself (personalization). The benefits of multiple measurement modalities can inform future decisions in digital biomarker research including data collection and wearable and mobile health device and application design. Of the 15 most important features, the overarching categories of circadian rhythm, demographics, diet, exercise, and stress were all represented, lending further credence to the usefulness of domain-driven feature engineering. There is ongoing debate about using HRV as a proxy measure of stress^90^ which may impact interpretation of the key model features. Additionally, the glucose management indicator (GMI) has been demonstrated to approximate HbA1c using continuous glucose monitoring data^91^. In future models, GMI may be able to replace the clinical HbA1c measurement, avoiding the need for this clinical measurement. Noninvasive glucose monitoring is a complex challenge, and our findings highlight the necessity of having multi-disciplinary teams in digital biomarker discovery research to integrate domain knowledge for domain-specific feature engineering.

In future work, we recommend expanding upon this study to improve glucose prediction using a personalized modeling approach with larger datasets and other machine learning methods. With a larger dataset, the integration of deep learning with the personalized approach may further increase the efficacy of non-invasively predicting glucose using wearables. In larger cohort studies, we recommend exploring age and body mass index and/or body fat percentage as covariates in the models. We also recommend the evaluation of this technology following the V3 (verification, analytical validation, and clinical validation) framework^29^. Here, we demonstrate the feasibility of using noninvasive methods for interstitial glucose classification and prediction. Follow up studies are needed to clinically evaluate this technology.

Glycemic health is at an all-time low: in the U.S., one in ten people have diabetes and one in three people have prediabetes; a dismal 20% of those with diabetes and 90% of those with prediabetes are undiagnosed^92^. In order to manage glucose fluctuations, it is important for patients to understand how their behaviors influence their blood glucose levels. There is a critical need for innovative, practical strategies to improve monitoring and management of glycemic health. In this study, we demonstrated the feasibility of using noninvasive and widely accessible mobile health and machine learning methods to non-invasively classify glucose excursions and predict glucose values.

## Methods

### Dataset recruitment and collection protocol

The study was approved by the Duke University Health System (DUHS) Institutional Review Board and written informed consent was obtained from all participants (Pro00101398). All subjects consented to the study and were compensated a total of $150 for their participation.

Patients (*N* = 16) were recruited for this prospective study from the Duke Endocrinology and Lipids Clinic through medical record review that identified patients between 35–65 years of age with high normal blood glucose (HbA1c 5.2–5.6) or prediabetes (HbA1c 5.7–6.4) (Supplementary Table 2). Exclusion criteria included cancer, COPD, cardiovascular disease, food allergies, or any antidiabetic drug use.

HbA1c was measured in the clinic on Day 0. Upon confirmation of A1C within range for this study, a continuous glucose monitor (CGM; Dexcom G6) and a non-invasive wearable smartwatch (Empatica E4) were worn continuously for 8–10 days (placement of study sensors shown in Fig. 1). A standardized breakfast meal with a high glycemic index (1.5 cups of Frosted Flakes and 1 cup Lactaid 2% Milk) was ingested every other morning during the monitoring period prior to ingesting any other food, drink, or medication, enabling repeated intraindividual monitoring of glycemic response. All other meals and snacks during the monitoring period were recorded through comprehensive written diet logging.

### Dataset

The Dexcom G6 records interstitial glucose concentration (mg/dL) every 5 min. The Empatica E4 contains four sensors: photoplethysmography (optical heart rate), electrodermal activity (galvanic skin response, related to sweat activity), skin temperature, and tri-axial accelerometry. Heart rate was recorded once per second, (calculated from photoplethysmography sampled at 64 Hz), electrodermal activity and skin temperature were recorded at 4 Hz, and accelerometry was recorded at 32 Hz.

In total, for this analysis we utilized over 25,000 interstitial glucose point measurements and 25,000 5-min epochs of wearable data measured over 8–10 days across 16 participants.

### Feature engineering

Features were engineered on data collected from the Empatica E4 wrist-worn wearable and the food diary. In total, 69 data-driven, domain-driven, and demographic historical features were used in developing the models, including features linked to diet, stress, exercise, circadian rhythm, and behavioral habits: all features known to contribute to blood glucose fluctuations (Supplementary Table 1). These features were computed every 5 min on a rolling basis (when appropriate, as described below). All features used in modeling were historical (5 min to 24 h prior to the measurement being predicted). Thus, the models were only given historical data to make predictions from.

Two of the 69 features were demographic data that incorporate user input into the model: biological sex and HbA1c. Another feature, the ‘personalization’ feature, differentiates each participant with a unique number so that models can learn relationships that may be individualistic.

Data-driven features for each of the 5-min intervals of smart watch data include 7 summary statistics for each sensor: mean, standard deviation, minimum, maximum, first quartile, third quartile, and skew. These summary statistics are computed for heart rate, accelerometry (vector magnitude of the three axes), electrodermal activity, and skin temperature.

For the domain-driven feature engineering, we focused on 4 factors that have demonstrated effects on blood glucose: stress, exercise (short term and long-term effects), circadian rhythm, and diet (including timing of meals, frequency of meals, and the short, medium, and long- term effects of protein, sugar, carbohydrates, and calories). We will break each of these down and explain the metrics calculated below:

The effects of stress have been measured physiologically for decades in psychology research, and we borrow some of their methods to quantify stress here^93–97^. Using the electrodermal activity data, we detected peaks and determined their prominences using the SciPy library in Python^98^. We required a distance between peaks of 1 second (4 data points) and a prominence of 0.3 micro-siemens to be considered a ‘unique peak’. We determined the number of peaks in each 5-min interval. This data was then aggregated using a rolling window approach: for the previous 2 h, on a rolling window, we determined the total number of peaks and the average number of peaks in each 5-min interval.

Heart rate variability (HRV) has also been demonstrated to fluctuate relating to both acute and chronic stress^90^. We utilized the inter-beat-interval data derived from the PPG and calculated 8 HRV metrics over each 5-min interval of data. Calculated metrics include mean HRV, median HRV, maximum HRV, minimum HRV, the standard deviation of intervals (SDNN), the root mean square of successive differences in the intervals (RMSSD), the number of successive intervals that differ by more than 50 ms (NN50), and the proportion of NN50 divided by the total number of intervals (pNN50). To calculate these metrics, we utilized open source code available in the Digital Biomarker Discovery Pipeline^32^ that has been validated against the state-of-the-art Kubios ECG software.

There have been shown to be both short-term and long-term effects of exercise on blood glucose levels^58,59^, which we wanted to examine using both accelerometry and heart rate data. While it is difficult to quantify “exercise” without user-input, we can determine when participants are being more active. Thus, we developed a calculation for “activity bouts”, or bouts of activity.

To calculate an “activity bout”, we took the mean accelerometry vector magnitude and the mean heart rate over the 5-min interval and compared it to the average of the prior historical data from the individual. If both the mean accelerometry and mean heart rate values for the 5-min interval were above the previous average, that interval is said to be an “activity bout”. In order to account for the short- and longer- term effects of activity on the glucose metabolism, we used a rolling window to compute the total activity bouts in the last hour and the average activity bouts in the previous 24 h. Additionally, we found the mean and maximum accelerometry vector magnitude over the previous two hours using a rolling window approach.

Circadian rhythm is a confounding variable in most physiology and it is important to take this into account as a feature, especially because there is a known connection between circadian rhythm and blood glucose^37^. We calculated the ‘minutes from midnight’ and the ‘hours from midnight’ as features indicative of circadian rhythm.

The time when an individual wakes can affect their circadian rhythm^99^. Heart rate and accelerometry have been used previously to determine waking versus sleep states^32^. For each participant and for each day, we examined when the accelerometry and heart rate mean and standard deviation were less than the average for that day. When two of the four measures were less than the average for the day, we assigned that interval a ‘0’. All other intervals were assigned a ‘1’. This data was then averaged over 3 h using a rolling window approach. We assigned the ‘Wake Time’ to when the slope of this data sharply changed and remained consistently higher 25 and 75 min after the wake time.

Diet is known to have strong effects on glucose metabolism. Here, we determined features explaining the short-, medium-, and long-term effects of food on glucose. We used a rolling window to sum the number of calories, grams of protein, grams of carbohydrates, and grams of sugar over three windows: 2 h, 8 h, and 24 h.

Each time a participant consumed a unique meal, snack, or caloric beverage, we assigned that interval a binary ‘1’. This allows us to determine timing of meals and how that, in conjunction with the circadian rhythm features, may affect the glucose metabolism. We then took a rolling sum of how many times an individual consumed over 2 h, 8 h, and 24 h. We also looked at the average amount of times an individual consumed over the 2-h, 8-h, and 24-h rolling windows.

### Developing personalized definitions of interstitial glucose excursions for detecting intraindividual excursions

The traditional definitions of glucose values that are ‘too high/low’, or hyperglycemia and hypoglycemia, have been defined from populations of people with diabetes^13,19–24^. These previous thresholds are insufficient and require personalization^26^. Thus, we wanted to re-define this and create personalized “high” and “low” glucose thresholds for each participant at each point in time. All outliers in the dataset were examined in exploratory data analysis relative to the food logs and other features, in addition to surrounding glucose values to ensure they were within reasonable bounds. We found no outliers in our dataset that were not explained by other features. We defined personalized glucose excursions as “PersHigh” and “PersLow”. We used a rolling window approach: for a glucose value to be classified as PersHigh, it had to exceed one standard deviation above the mean for the last 24 h. To be considered PersLow, the value had to be below one standard deviation below the mean for the last 24 h. The third category, “PersNorm” had to fall within one standard deviation above or below the mean for the last 24 h. We tested the extent to which the distributions of these categories are normal using the Kolmogorov-Smirnov Test to test between our distribution and a normal distribution.

### Classification of glucose excursions

We balanced our classes of PersNorm, PersHigh, and PersLow for a total *N* = 8666 because the entire dataset (*N* = ~25,000) was highly imbalanced with the majority of data points being in the PersNorm category. Our model was implemented in a repeated stratified k-fold cross validation schema with 10 splits and 3 repeats. Within each fold, we implemented recursive feature selection to select the 20 most important features, which were used to train the model. We iterated through several estimator methods to determine the optimal method for the recursive feature elimination, including logistic regression, perceptron, decision tree, random forest, and gradient boosting classifier. We utilized the estimator that resulted in the highest accuracy for our final model, the decision tree estimator. Finally, for each fold, using the features selected with the recursive feature elimination, we trained a decision tree classifier.

In addition to the primary, cross-validated model, we developed a 70/30 train/test split decision tree classifier model and a logistic regression model.

We evaluated our model using balanced accuracy, weighted precision, weighted recall, and weighted f1 score, from the python package scikit-learn^100^ at each fold in the cross validation and report the mean and standard deviation of each metric. We also examined the amount of variance explained by the model by reporting R^2^.

### Glucose prediction

Using the same features derived above in our feature engineering, we developed predictive models for predicting actual interstitial glucose values from noninvasive wearables and food diary data. We developed both a population model with LOPOCV and a personalized model trained and tested on each participant’s own data. We utilized a dataset of over 25,000 glucose measurements and 25,000 5-min intervals of smart watch data measured over 8–10 days across 16 participants. There were an average of 1500 glucose measurements per participant.

We developed a regression gradient boosting decision-tree-based regression model using the XGBoost^101^ algorithm and used tuned hyperparameters for all models (maximum depth=6, number of estimators=100, learning rate = 0.1). The prediction target of our models was interstitial glucose at every 5-min interval. The model was trained using the 69 engineered features and feature selection (cutoff = 0.005) was performed with a random forest regression model (1000 trees) using impurity-based feature importances for each fold of our LOPOCV. Feature importance was taken at each fold in our LOPOCV model and averaged to determine the most important features in predicting glucose.

For the population, LOPOCV model, we iterated over each participant (fold), using all other participants as the ‘training set’ and using each participant as the ‘test set’. For the personalized model, we trained on the first contiguous half (50%) of the participant’s data and tested on the remaining half of the participant’s data.

Models were evaluated using root mean squared error (RMSE), mean average percent error (MAPE), and accuracy (100-MAPE) for each fold of our LOPOCV models. We report the mean and standard deviation over the population of these evaluation metrics.

### Analysis of features

In order to determine important features for predicting glucose, we trained a random forest regression model (1000 trees) with LOPOCV and averaged impurity-based feature importances for each fold of our LOPOCV to determine the most important features across participants.

Features were aggregated together into the following categories: ‘food’, ‘circadian rhythm’, ‘stress’, ‘activity’, ‘temperature’, ‘heart rate’, ‘electrodermal activity’, ‘biological sex’, ‘HbA1c’, and ‘personalization’. They were further categorized by the source of the data: ‘food log’, ‘wearable’, ‘user input’, and ‘model’. We also divided the features into categories based on how the feature engineering was performed: ‘data-driven’, ‘domain-driven’, and ‘other’. Feature importances across these categories were aggregated by averaging the importance across each fold in our LOPOCV random forest regression model. We determined the percent importance of each of these feature categories out of the total feature importance; thus, we report importance as a percent when evaluating the features.

### Reporting summary

Further information on research design is available in the Nature Research Reporting Summary linked to this article.

## Supplementary information

Supplementary Information

Reporting Summary

## Data Availability

The data sets generated during and/or analyzed during the current study will be submitted one year from the publication date to the Digital Health Data Repository in the Digital Biomarker Discovery Pipeline.

## Code availability

The code generated for this study are available open-access in the Digital Biomarker Discovery Pipeline (dbdp.org).
