# Supplementary material for: Engineering digital biomarkers of interstitial glucose from noninvasive smartwatches
Source: NPJ Digit Med. 2021 Jun 2;4:89. doi: 10.1038/s41746-021-00465-w (PMC8172541; doi:10.1038/s41746-021-00465-w)
Supplement: Supplementary file 1 — Supplementary Information [file 41746_2021_465_MOESM1_ESM.pdf]

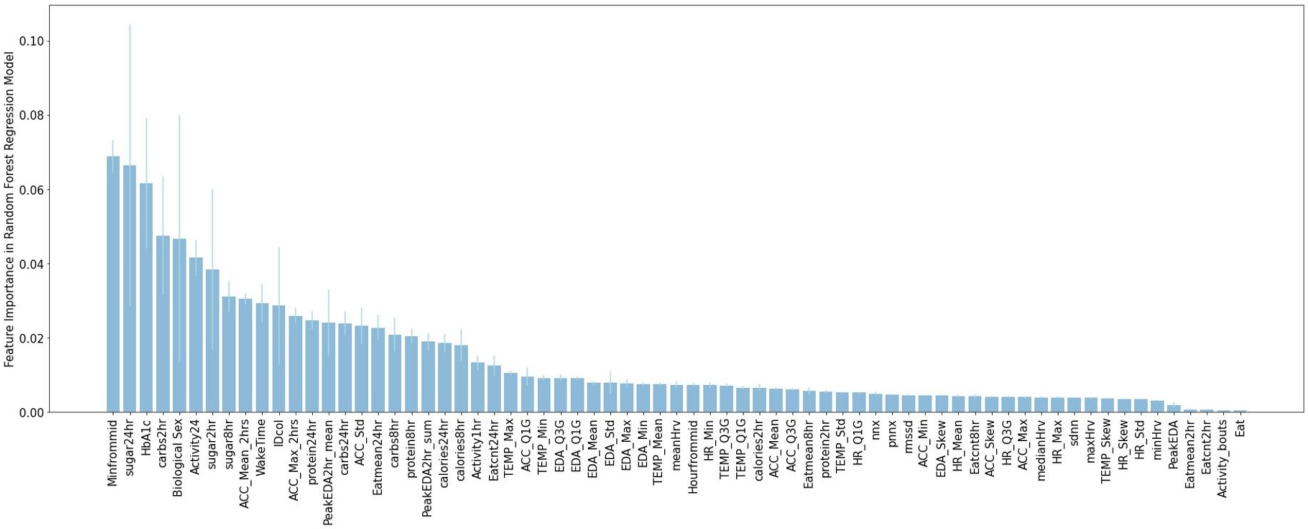

Supplementary Figure 1. Bar plot of importance of 69 domain-driven and data-driven features in glucose predictions. Importance was determined from a random forest feature selection model using impurity-based features.

Supplementary Table 1.

The 69 data-driven and domain-driven features engineered for glucose prediction and classification, the type of feature, the source of the feature, and the calculation/description of the derivation.

Feature Name	Feature Type	Source	Calculation/Description
Biological Sex	Demographics	User-defined	N/A
HbA1c	Clinical Metrics	User-defined	N/A
EDA_Mean	Electrodermal Activity	Wearable	$\mu = \frac{\sum_N \overline{x_i}}{N}$<br>N= number of points in a 5-minute interval unless otherwise designated
EDA_Std	Electrodermal Activity	Wearable	$\sigma = \sqrt{\frac{\sum (x_i - \mu)^2}{N}}$
EDA_Min	Electrodermal Activity	Wearable	$minimum(x_i\ over\ N\ interval)$
EDA_Max	Electrodermal Activity	Wearable	$maximum(x_i\ over\ N\ interval)$
EDA_Q1G	Electrodermal Activity	Wearable	$first\ quartile(x_i\ over\ N\ interval)$

EDA_Q3G	Electrodermal Activity	Wearable	$third\ quartile(x_i\ over\ N\ interval)$
EDA_Skew	Electrodermal Activity	Wearable	$\frac{\sum_i^N (x_i - \bar{x})^3}{(N-1) * \sigma^3}$
HR_Mean	Heart Rate	Wearable	$\mu = \frac{\sum_N \bar{x_i}}{N}$ N= number of points in a 5-minute interval
HR_Std	Heart Rate	Wearable	$\sigma = \sqrt{\frac{\sum (x_i - \mu)^2}{N}}$
HR_Min	Heart Rate	Wearable	$minimum(x_i\ over\ N\ interval)$
HR_Max	Heart Rate	Wearable	$maximum(x_i\ over\ N\ interval)$
HR_Q1G	Heart Rate	Wearable	$first\ quartile(x_i\ over\ N\ interval)$
HR_Q3G	Heart Rate	Wearable	$third\ quartile(x_i\ over\ N\ interval)$
HR_Skew	Heart Rate	Wearable	$\frac{\sum_i^N (x_i - \bar{x})^3}{(N-1) * \sigma^3}$
TEMP_Mean	Skin Temperature	Wearable	$\mu = \frac{\sum_N \bar{x_i}}{N}$ N= number of points in a 5-minute interval
TEMP _Std	Skin Temperature	Wearable	$\sigma = \sqrt{\frac{\sum (x_i - \mu)^2}{N}}$
TEMP _Min	Skin Temperature	Wearable	$minimum(x_i\ over\ N\ interval)$
TEMP _Max	Skin Temperature	Wearable	$maximum(x_i\ over\ N\ interval)$
TEMP _Q1G	Skin Temperature	Wearable	$first\ quartile(x_i\ over\ N\ interval)$
TEMP _Q3G	Skin Temperature	Wearable	$third\ quartile(x_i\ over\ N\ interval)$
TEMP _Skew	Skin Temperature	Wearable	$\frac{\sum_i^N (x_i - \bar{x})^3}{(N-1) * \sigma^3}$
ACC_Mean	Accelerometry	Wearable	$\mu = \frac{\sum_N \bar{x_i}}{N}$ N= number of points in a 5-minute interval
ACC _Std	Accelerometry	Wearable	$\sigma = \sqrt{\frac{\sum (x_i - \mu)^2}{N}}$
ACC _Min	Accelerometry	Wearable	$minimum(x_i\ over\ N\ interval)$
ACC _Max	Accelerometry	Wearable	$maximum(x_i\ over\ N\ interval)$
ACC _Q1G	Accelerometry	Wearable	$first\ quartile(x_i\ over\ N\ interval)$
ACC _Q3G	Accelerometry	Wearable	$third\ quartile(x_i\ over\ N\ interval)$
ACC _Skew	Accelerometry	Wearable	$\frac{\sum_i^N (x_i - \bar{x})^3}{(N-1) * \sigma^3}$
PeakEDA	Stress	Wearable	$\sum_i^N peaks$ Peaks defined by rolling peak detection (height=0, distance=4, prominence=0.3)
PeakEDA2hr_sum	Stress	Wearable	$\sum_i^{N_{2\ hours}} PeakEDA$ *rolling calculation

PeakEDA2hr_mean	Stress	Wearable	$\frac{\sum_i^{N_{2\ hours}} PeakEDA}{N_{2\ Hours}}$
maxHRV	Stress	Wearable	$maximum(IBI_i\ over\ N\ interval)$
minHRV	Stress	Wearable	$minimum(IBI_i\ over\ N\ interval)$
medianHRV	Stress	Wearable	$median(IBI_i\ over\ N\ interval)$
meanHRV	Stress	Wearable	$\frac{\sum_N \overline{IBI_i}}{N}$
SDNN	Stress	Wearable	$\sqrt{\sigma^2}$ of IBI over N interval
NN50	Stress	Wearable	$\sum_i^N when\ \lvert IBI[i+1] - IBI[i] \rvert > 50$
pNN50	Stress	Wearable	$\frac{NN50}{len(N)}$
RMSSD	Stress	Wearable	$\frac{\sqrt{\sum_i^N\ (\lvert IBI[i+1] - IBI[i] \rvert)^2}}{len(N)}$
calories2hr	Diet	Food Log	$\sum_i^{N_{2\ Hours}} x_i$ *rolling calculation
protein2hr	Diet	Food Log	$\sum_i^{N_{2\ Hours}} x_i$ *rolling calculation
sugar2hr	Diet	Food Log	$\sum_i^{N_{2\ Hours}} x_i$ *rolling calculation
carbs2hr	Diet	Food Log	$\sum_i^{N_{2\ Hours}} x_i$ *rolling calculation
calories8hr	Diet	Food Log	$\sum_i^{N_{8\ Hours}} x_i$ *rolling calculation
protein8hr	Diet	Food Log	$\sum_i^{N_{8\ Hours}} x_i$ *rolling calculation
sugar8hr	Diet	Food Log	$\sum_i^{N_{8\ Hours}} x_i$ *rolling calculation
carbs8hr	Diet	Food Log	$\sum_i^{N_{8\ Hours}} x_i$ *rolling calculation
calories24hr	Diet	Food Log	$\sum_i^{N_{24\ Hours}} x_i$ *rolling calculation

protein24hr	Diet	Food Log	$\sum_{i}^{N_{24\,Hours}} x_i$<br>*rolling calculation
sugar24hr	Diet	Food Log	$\sum_{i}^{N_{24\,Hours}} x_i$<br>*rolling calculation
carbs24hr	Diet	Food Log	$\sum_{i}^{N_{24\,Hours}} x_i$<br>*rolling calculation
Eat	Diet	Food Log	binary: 0 if not eating currently, 1 if currently eating
Eatcnt2hr	Diet	Food Log	$\sum_{i}^{N_{2\,Hours}} x_i$<br>*rolling calculation
Eatcnt8hr	Diet	Food Log	$\sum_{i}^{N_{8\,Hours}} x_i$<br>*rolling calculation
Eatcnt24hr	Diet	Food Log	$\sum_{i}^{N_{24\,Hours}} x_i$<br>*rolling calculation
Eatmean2hr	Diet	Food Log	$\frac{\sum_{N_{2\,Hours}} \overline{x_i}}{N_{2\,Hours}}$<br>* rolling calculation
Eatmean8hr	Diet	Food Log	$\frac{\sum_{N_{8\,Hours}} \overline{x_i}}{N_{8\,Hours}}$<br>* rolling calculation
Eatmean24hr	Diet	Food Log	$\frac{\sum_{N_{24\,Hours}} \overline{x_i}}{N_{24\,Hours}}$<br>* rolling calculation
WakeTime	Circadian Rhythm	Wearable	1. One point assigned based on whether HR_Mean, HR_Std, ACC_Mean, and ACC_Std for each N interval are each less than historical average.<br>2. If points >2, binary assignment of 1<br>3. Data averaged over 3 hours using rolling approach<br>4. Wake Time assigned when slope of data changed and remained consistently higher for 25 and 75 minutes after the designated Wake Time
Minfrommid	Circadian Rhythm	Wearable	Minutes from midnight
Hourfrommid	Circadian Rhythm	Wearable	Hours from midnight
ACC_mean_2hrs	Activity/exercise	Wearable	$\frac{\sum_{N_{2\,Hours}} \overline{x_i}}{N_{2\,Hours}}$<br>* rolling calculation
ACC_max_2hrs	Activity/exercise	Wearable	$maximum(x_i\ over\ N = 2\ Hours)$<br>* rolling calculation

Activity_bouts	Activity/exercise	Wearable	Binary exercise point designated if both ACC_Mean and HR_Mean over N interval were greater than historical mean
Activity24	Activity/exercise	Wearable	$\frac{\sum_{N_{24\,Hours}} \overline{Activity\_bouts_i}}{N_{24\,Hours}}$ * rolling calculation
Activity1hr	Activity/exercise	Wearable	$\sum_{N\ 1\ hour} Activity\_bouts_i$ * rolling calculation
ID	Personalization	Model	Assigned ID to designate different participants

Supplementary Table 2. Demographics of Study Participants

**Characteristic**		**Number of Participants**
**Sex**		
	**Male**	8
	**Female**	8
**Age, years**		
	**0-34**	0
	**35-39**	0
	**40-49**	1
	**50-59**	11
	**60-69**	4
	**70+**	0
**Race**		
	**White/Caucasian**	11
	**Black/African American**	4
	**Biracial**	1
	**Other**	0
