# Supplementary material for: Engineering digital biomarkers of interstitial glucose from noninvasive smartwatches
Source: NPJ Digit Med. 2021 Jun 2;4:89. doi: 10.1038/s41746-021-00465-w (PMC8172541; doi:10.1038/s41746-021-00465-w)
Supplement: Supplementary file 2 — Reporting Summary [file 41746_2021_465_MOESM2_ESM.pdf]

nature research

Corresponding author(s): Jessilyn P. Dunn

Last updated by author(s): Apr 28, 2021

Reporting Summary

Nature Research wishes to improve the reproducibility of the work that we publish. This form provides structure for consistency and transparency in reporting. For further information on Nature Research policies, see our Editorial Policies and the Editorial Policy Checklist.

Statistics

For all statistical analyses, confirm that the following items are present in the figure legend, table legend, main text, or Methods section.

n/a	Confirmed	
☐	☒	The exact sample size (*n*) for each experimental group/condition, given as a discrete number and unit of measurement
☐	☒	A statement on whether measurements were taken from distinct samples or whether the same sample was measured repeatedly
☐	☒	The statistical test(s) used AND whether they are one- or two-sided<br>*Only common tests should be described solely by name; describe more complex techniques in the Methods section.*
☐	☒	A description of all covariates tested
☐	☒	A description of any assumptions or corrections, such as tests of normality and adjustment for multiple comparisons
☐	☒	A full description of the statistical parameters including central tendency (e.g. means) or other basic estimates (e.g. regression coefficient) AND variation (e.g. standard deviation) or associated estimates of uncertainty (e.g. confidence intervals)
☒	☐	For null hypothesis testing, the test statistic (e.g. *F*, *t*, *r*) with confidence intervals, effect sizes, degrees of freedom and *P* value noted<br>*Give P values as exact values whenever suitable.*
☒	☐	For Bayesian analysis, information on the choice of priors and Markov chain Monte Carlo settings
☒	☐	For hierarchical and complex designs, identification of the appropriate level for tests and full reporting of outcomes
☒	☐	Estimates of effect sizes (e.g. Cohen's *d*, Pearson's *r*), indicating how they were calculated

Our web collection on statistics for biologists contains articles on many of the points above.

Software and code

Policy information about availability of computer code

Data collection	Data collection was performed using Empatica E4 reporting software and Dexcom G6 reporting software.
Data analysis	Software utilized in the pre-processing, exploratory data analysis, and feature engineering for this study are available open-source in the Digital Biomarker Discovery Pipeline (dbdp.org).

For manuscripts utilizing custom algorithms or software that are central to the research but not yet described in published literature, software must be made available to editors and reviewers. We strongly encourage code deposition in a community repository (e.g. GitHub). See the Nature Research guidelines for submitting code & software for further information.

Data

Policy information about availability of data

All manuscripts must include a data availability statement. This statement should provide the following information, where applicable:

- Accession codes, unique identifiers, or web links for publicly available datasets
- A list of figures that have associated raw data
- A description of any restrictions on data availability

The data sets generated during and/or analyzed during the current study will be submitted one year from the publication date to the Digital Health Data Repository in the Digital Biomarker Discovery Pipeline.

Field-specific reporting

Please select the one below that is the best fit for your research. If you are not sure, read the appropriate sections before making your selection.

☒ Life sciences ☐ Behavioural & social sciences ☐ Ecological, evolutionary & environmental sciences

For a reference copy of the document with all sections, see nature.com/documents/nr-reporting-summary-flat.pdf

Life sciences study design

All studies must disclose on these points even when the disclosure is negative.

Sample size	The sample size of 25,000 samples for the glucose prediction models is sufficient to train the traditional machine learning methods for this feasibility study.
Data exclusions	No data was excluded from these analyses.
Replication	Several models were built for each use case and cross validation (leave one person out cross validation or k-fold cross validation) was performed to ensure accuracy of models across participants.
Randomization	This is not relevant to our study because there was not an intervention.
Blinding	Group allocation was not relevant to our study because there was not an intervention.

Reporting for specific materials, systems and methods

We require information from authors about some types of materials, experimental systems and methods used in many studies. Here, indicate whether each material, system or method listed is relevant to your study. If you are not sure if a list item applies to your research, read the appropriate section before selecting a response.

Materials & experimental systems

n/a	Involved in the study	
☒	☐	Antibodies
☒	☐	Eukaryotic cell lines
☒	☐	Palaeontology and archaeology
☒	☐	Animals and other organisms
☐	☒	Human research participants
☒	☐	Clinical data
☒	☐	Dual use research of concern

Methods

n/a	Involved in the study	
☒	☐	ChIP-seq
☒	☐	Flow cytometry
☒	☐	MRI-based neuroimaging

Human research participants

Policy information about studies involving human research participants

Population characteristics	16 total (8 female), aged 40-69, 11 white/caucasian; 4 African American; 1 Bi-racial participant (all demographics are available in Supplementary Table 2)
Recruitment	Patients (N=16) were recruited for this prospective study from the Duke Endocrinology and Lipids Clinic through medical record review that identified patients between 35-65 years of age with high normal blood glucose (HbA1c 5.2-5.6) or prediabetes (HbA1c 5.7-6.4). Exclusion criteria included cancer, COPD, cardiovascular disease, food allergies, or any antidiabetic drug use.
Ethics oversight	Duke University Health System (DUHS) Institutional Review Board (Pro00101398)

Note that full information on the approval of the study protocol must also be provided in the manuscript.
